# Comparison the inflammatory effects of early supplemental parenteral nutrition plus enteral nutrition versus enteral nutrition alone in critically ill patients

**Published:** 2010

**Authors:** R. Abrishami, A. Ahmadi, M. Abdollahi, A. Moosivand, H. Khalili, A. Najafi, K. Gholami, H. Hamishehkar, A. Peivandi Yazdi, M. Mojtahedzadeh

**Affiliations:** 1 Faculty of Pharmacy; 2 School of Medicine, Tehran University of Medical Sciences, Tehran; 3 Department of Clinical Pharmacy, Faculty of Pharmacy, Tabriz University of Medical Sciences, Tabriz; 4 Department of Anesthesia and Critical Care, Mashhad University of Medical Sciences, Mashhad, Iran

**Keywords:** Enteral nutrition, Parenteral nutrition, Inflammation

## Abstract

**Background and the purpose of the study:**

It is believed that enteral nutrition (EN) support is the preferred route as compared to parenteral nutrition (PN). Critically ill patients on EN receive less than 60% of their metabolic requirements. To meet patients’ calorie goal addition of PN to EN was proposed. This study was conducted to determine whether supplemental PN have any difference with EN alone in regard to inflammatory indices.

**Methods:**

Twenty patients were randomized to either receive EN alone or EN+PN for 7 days. Pre albumin and inflammatory indices including interleukin IL-1, IL-6 and tumor necrosis factor-α (TNF-α) were measured on days of 0, 3,7. Also Sequential Organ Failure Assessment (SOFA) score and Therapeutic Intervention Scoring System-28 (TISS-28) score were calculated on days of 0, 3 and 7.

**Results and major conclusion:**

IL-1, IL-6 and TNF-α did not show significant difference between two interventions. Pre-albumin was increased from baseline by 9% and 81% in EN and EN+PN groups respectively but it did not reach to statistical significance. SOFA score did not show significant difference. TISS score was higher in EN+PN group on days of 3 and 7.

No difference was found between EN and EN+PN regimens in regard to inflammation, while severity of illness may not change with these regimens, nursing workload increases with implementation of supplemental PN.

## INTRODUCTION

Providing nutrition to critically ill patients is being considered as a primary therapeutic strategy (1). It is believed that enteral nutrition (EN) support is the preferred route as compared with parenteral nutrition (PN) (2). Malnutrition has been documented in the critically ill patients (3). Often achieving caloric goals with EN is not feasible (4) and critically ill patients on EN receive less than 60% of their metabolic requirements (5). To meet calorie goal addition of PN to EN has been proposed. There are few clinical trials that have compare EN versus EN+PN. There is no report on any trial to compare inflammation between EN and EN+PN, and effects of inflammation on patient's disease course and outcome (6, 7). In this study, inflammatory parameters of EN and EN+PN regimens during the first week of nutritional support in the ICU were compared.

## MATERIALS AND METHODS

This study was a randomized, controlled clinical trial carried out in a 10 bed ICU of Sina teaching hospital (Tehran, Iran) approved by institute ethic board. Written consent forms obtained from patients’ relatives.

### 

#### Patients

Two groups of 10 patients were enrolled between November 2007 and May 2009. Patients eligible for inclusion were those of 18 years old or over, recent ICU admission (<24 hrs), having systemic inflammatory response syndrome (SIRS), and Acute Physiology and Chronic Health Evaluation II (APACHE II) score greater than 10 and expected not to feed via oral route for at least 5 days (8, 9). Patients with high probability of death in the next 7 days of admission, pregnant, lactating, and having EN contraindication were excluded from the study. All patients received routine ICU care.

#### Nutritional support

##### Enteral (EN)

Patients were fed via naso-gastric (NG) tube with Fresubin® Original (Fresenius Kabi, Germany) a solution with 1 kCal/ml energy. An average 70 kg patient received 50 ml of this solution initially every 3 hrs which increased with 50 ml increments to a maximum of 300 ml every 3 hrs with a rate of 100 ml/h. Prior to the next feeding, residual volume was checked and if it was greater than 300 ml feeding was delayed by 3 hrs and metoclopramide was administered.

#### Enteral plus Parenteral (EN+PN)

PN consisted of 500 ml of 10% amino acid solution (B Braun, Germany), and 500 mL of 50% dextrose solution (Samen, Iran) infused over 24 hrs. Enteral support in this group was the same as EN group.

#### Assessment

The patient's demographic data, cause of admission, APACHE II score and serum albumin were recorded on admission (day 0). Sequential Organ Failure Assessment (SOFA) score and TISS-28 score were calculated on days of 0, 3 and 7 to evaluate severity of illness and to determine number of interventions, respectively (10, 11). Length of ICU and hospital stay were recorded. Venous blood samples were taken just prior to beginning of and then 3 and 7 days after initiation of nutrition support. The serum of each sample was stored at -80°C for subsequent analysis of inflammatory cytokines including IL-1, IL-6 tumor necrosis factor-α (TNF-α) and pre-albumin. The cytokines were measured using enzyme linked immunosorbent assay (ELISA) kits (Bender MedSystems, Austria). Pre-albumin was measured using radial immuno-diffusion technique (The Binding Site, United Kingdom).

#### Statistical analyses

Statistical analysis was performed using SPSS- 11.5 software (SPSS Inc., Chicago, USA). Non-parametric data were analyzed using Mann-Whitney test. Normally distributed data were analyzed using repeated measures analysis of variances and student t-test. *P* values less than 0.05 was considered significant.

## RESULTS AND DISCUSSION

From 20 patients that were entered in the study (Table 1), one patient in EN+PN group and two patients in EN group died before the day of 7, and one patient in EN+PN group was transferred to another hospital on the third day. No significant differences were found in age, severity scores, mean arterial pressure, blood pH, and serum albumin levels of the patients between two groups on admission.

**Table 1:** Demographic and baseline characteristics of the patients.

Parameter	Mean±SE		*p value*

	EN	EN+PN	
Age (years)	58.40±5.07	54.90±5.16	0.63
APACHE II score	17.00*	18.50*	–
SOFA score	9.00*	7.00*	–
Mean Arterial Pressure (MAP) (mm Hg)	76.30±3.36	80.10±6.13	0.59
Blood pH	7.36±0.03	7.35±0.04	0.75
Albumin (g/dL)	3.05±0.27	2.98±0.20	0.83
Trauma admission	3	4	–
Surgical admission	4	4	–
Medical admission	3	2	–

* Numbers represent median.

**Figure 1:**
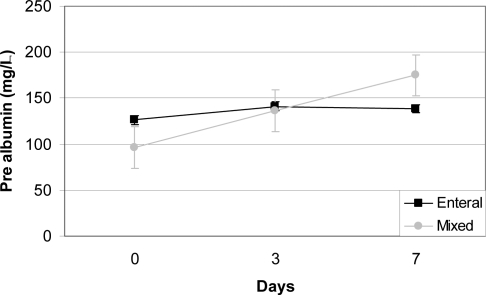
Patients’ serum pre-albumin levels in enteral and enteral plus parenteral groups. Differences between groups were not significant (*p*>0.05). Figure shows mean pre-albumin levels±SE.

The patients serum pre-albumin was increased from baseline by 9% and 81% in EN and EN+PN groups respectively but it did not reach to statistical significance (*p*=0.658; Figure 1).

Results showed that patients who received EN+PN received more energy than patients on EN alone (12, 13). EN+PN corrects pre-albumin faster than EN or PN (14). Increased serum pre-albumin concentration in EN+PN group may be related to the increase in calorie intake with PN supplementation.

**Figure 2:**
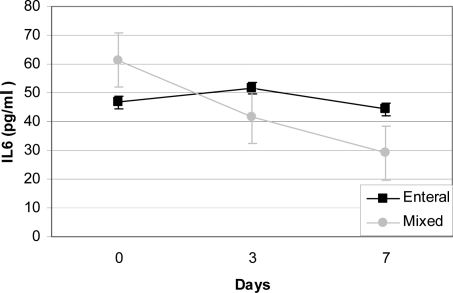
Interleukin 6 levels in enteral and enteral plus parenteral groups. Differences between groups were not significant (*p*>0.05). Figure shows mean interleukin 6 levels±SE.

Levels of IL-6 did not change significantly from baseline up to day7 (*p*>0.05; Figure 2), but its value declined by 52% and 5% in EN+PN and EN group, respectively. TNF-α values decreased by 4.3% from baseline in EN group and increased by 5.1% in EN+PN group on the day of 7 (*p*>0.05). IL-1 concentrations on the day of 7 increased by 3% in EN group and decreased by 5.6% in EN+PN group from baseline (*p*>0.05).

In contrast to reports that TNF is significantly lower in EN than TPN patients (15) and malnourished patients maintain their capacity of releasing inflammatory mediators (16), another study has shown that TNF and IL-6 serum levels were not different between patients on prolonged home PN and healthy volunteers (17).

IL-6 is an independent outcome predictor in the ICU (18) and in this study IL-6 levels decreased in EN+PN group, but it did not reach to statistical significance which may be due to small sample size. Different results of this study may be due to different study population.

In agreement with results of other investigation (12, 14, 19, 20) daily SOFA score in this study showed no different mortality rate in patients with EN+PN compared to those who only received EN.

In a systematic review of five clinical trials comparing EN versus EN+PN, no significant differenc on mortality were reported and it was concluded that in well-nourished patients with intact GI tract, supplementing PN to EN has no clinical benefit (21). Route of energy delivery may not affect patient outcome, and delivering enough energy and substrate to hypercatabolic critically ill patients may be more important. Higher demands of these patients must be matched with an appropriate supply. However increased mortality in some patients especially severally burned patients (22) has been reported (23).

TISS score did not show significant difference on the day of 0 (*p*=0.133), but on days of 3 and 7 two groups showed significant difference (*p*=0.03). TISS scores were higher in EN+PN group (35.40 vs.39.30 on day of 3 and 36.50 vs. 39.38 on day of 7).

Bauer et al used OMEGA score to assess the burden of care and reported no difference in patients on either EN or EN+PN (14). Higher scores in EN+PN group may be related to higher nursing workload. Considering that each TISS-28 point corresponds to 10.6 minutes of the work time of a nurse (24), a nurse caring for a patient on EN+PN spent about 30 minutes higher than caring for a patient on EN alone. This may be due to the time spent for preparation of PN administration and related cares.

Mean length of hospitalization of the patients were 36.50 and 37.40 days in EN and EN+PN groups respectively (*p*=0.917). The mean length of the patient stays in the ICU was 27.70 in EN and 25.70 days in EN+PN group (*p*=0.785).

Similarly Huang et al have reported no difference in ICU and hospital length of stays (25). However Deegan showed that patients who are on EN+PN had prolonged length of stay in comparison with those on EN alone (12).

## CONCLUSION

No difference was found between EN and EN+PN regimens in regard to effects on inflammatory responses. Severity of illness may not change with these regimens. Nursing workload increases with implementation of supplemental PN. Until sufficient data from large randomized clinical trials is available using EN with parenteral supplementation is not recommended.

## References

[1] McClave SA, Heyland DK, FINK M.P (2005). Critical care nutrition. Textbook of critical care.

[2] Heyland DK, Cook D, Guyatt G (1993). Enteral nutrition: A critical appraisal of the evidence. Intensive Care Medicine.

[3] Jonghe BD, Appere-De-Vechi C, Tran MFB, Merrer J, Melchior J-C, Outin H (2001). A prospective survey of nutritional support practices in intensive care unit patients: what is prescribed? What is delivered?. Critiacl Care Medicine.

[4] Reid C (2006). Frequency of under- and overfeeding in mechanically ventilated ICU patients: causes and possible consequences. J. Hum. Nutr Dietet.

[5] Heyland DK, Schroter-Noppe D, Drower J, Jain M, Keefe L, Dhaliwal R, Day A (2003). Nutrition support in the critical care setting: current practices in Canadian ICUs-opportunities for I provement?. Journal of Parenteral and Enteral Nutrition.

[6] Forasassi C, Golmard J-L, Pautas E, Piette Fo, Myara I, Raynaud-Simon A (2009). Inflammation and disability as risk factors for mortality in elderly acute care patients. Archives of Gerontology and Geriatrics.

[7] Hadidi E, Mojtahedzadeh M, Paknejad M, Nikfar S, Zamani M, Sahraian M, Eftekhar B (2006). Alterations of blood IL-8, TGF-β1 and nitric oxide levels in relation to blood cells in patients with acute brain injury. Therapy.

[8] Knaus W, Draper E, Wagner D, Zimmerman J (1985). APACHE II: A severity of disease classification system. Critical Care Medicine.

[9] Society of Critical Care Medicine Consensus Conference Committee: American College of Chest Physicians/ Society of Critical Care Medicine Consensus Conference: definitions for sepsis and organ failure and guidelines for the use of innovative therapies in sepsis (1992). Critical Care Medicine.

[10] Vincent J, Moreno R, Takala J, Matos R (1996). The SOFA (Sepsis-related Organ Failure Assessment) score to describe organ dysfunction/failure. On behalf of the Working Group on Sepsis-Related Problems of the European Society of Intensive Care Medicine. Intensive Care Medicine.

[11] Moreno R, Vincent J.-L, Matos R, Mendonça A, Cantraine F, Thijs L, Takala J, Sprung C, Antonelli M, Bruining H, Willatts S (1999). The use of maximum SOFA score to quantify organ dysfunction/failure in intensive care. Results of a prospective, multicentre study. Working Group on Sepsis related Problems of the ESICM. Intensive Care Medicine.

[12] Deegan H, Dent S, Keefe L, Drover J, Heyland D (1999). Supplemantal parenteral nutrition in the critically ill patient: a retrospective study. Clinical Intensive Care.

[13] Villet S, Chiolero RL, Bollmann MD, Revelly J-P, RN M-CC, Delarue J, Berger MM (2005). Negative impact of hypocaloric feeding and energy balance on clinical outcome in ICU patients. Clinical Nutrition.

[14] Bauer P, Charpentier C, Bouchet C, Nace L, Raffy E, Gacconet N (2000). Parenteral with enteral nutrition in the critically ill. Intensive Care Medicine.

[15] Chen Z, Wang S, Yu B, Li A (2007). A comparison study between early enteral nutrition and parenteral nutrition in severe burn patients. Burns.

[16] Delgado AF, Okay TS, Leone C, Nichols B, Negro GMD, Vaz FAC (2008). Hospital malnutrition and inflammatory response in critically ill children and adolescents admitted to a tertiary intensive care unit. Clinics.

[17] Hise ME, Compher C, Harlan L, Kohlmeier JE, Benedict SH, Gajewsk B, Brown JC (2006). Inflammatory mediators and immune function are altered in home parenteral nutrition patients. Nutrition.

[18] Dimopoulou I, Orfanos S, Kotanidou A, Livaditi O, Giamarellos-Bourboulis E, Athanasiou C, Korovesi I, Sotiropoulou C, Kopterides P, Ilias I, Kanellakopoulou K, Armaganidis A (2008). Plasma pro- and anti- inflammatory cytokine levels and outcome prediction in unselected critically ill patients. Cytokine.

[19] Herndon D, Stein M, Rutan T, Abston S, Linares H (1987). Failure of TPN supplementation to improve liver function, immunity, and mortality in thermally injured patients. The Journal of Trauma.

[20] Mojtahedzadeh M, Khalili H, Oveisi M, Tavakolli F, Abdollahi M (2003). Quality of nutrition in critically ill patients. Biomedical Research.

[21] Dhaliwal R, Jurewitsch B, Harrietha D, Heyland DK (2004). Combination enteral and parenteral nutrition in critically ill patients: harmful or beneficial? A systematic review of the evidence. Intensive Care Medicine.

[22] Herndon D, Barrow R, Stein M, Linares H, Rutan T, Abston RRS (1989). Increased mortality with intravenous supplemental feeding in severly burned patients. Journal of Burn Care & Rehabilitation.

[23] Dunham C, Frankenfield D, Belzburg H, Wiles C, Cushing B, Grant Z (1994). Gut failure-Predictor of or contributor to mortality in mechanically ventilated blunt trauma patients?. The Journal of Trauma.

[24] Miranda D, Rijk Ad, Schaufeli W (1996). Simplified Therapeutic Intervention Scoring System: the TISS-28 items – results from a multicenter study. Critical Care Medicine.

[25] Huang YC, Yen CE, Cheng CH, Jih KS, Kan MN (2000). Nutritional status of mechanically ventilated critically ill patients: comparison of different types of nutritional support. Clinical Nutrition.

